# Effect of region of interest size on ADC measurements in pancreatic adenocarcinoma

**DOI:** 10.1186/s40644-017-0116-6

**Published:** 2017-05-02

**Authors:** Chao Ma, Xiaoyu Guo, Li Liu, Qian Zhan, Jing Li, Chengcheng Zhu, Li Wang, Jing Zhang, Xu Fang, Jianxun Qu, Shiyue Chen, Chengwei Shao, Jian-ping Lu

**Affiliations:** 1 Department of Radiology, Changhai Hospital of Shanghai, the Second Military Medical University, No.168 Changhai Road, 200433 Shanghai, China; 2 Department of Radiology and Biomedical Imaging, University of California, San Francisco, California USA; 3 Department of Pathology, Changhai Hospital of Shanghai, the Second Military Medical University, Shanghai, China; 4 GE Healthcare, MR Group, Shanghai, China

**Keywords:** DWI, ADC, Pancreatic cancer, Region of interest, MRI

## Abstract

**Background:**

To investigate the influence of region of interest (ROI) size on tumor apparent diffusion coefficient (ADC) measurements in pancreatic cancer.

**Methods:**

The study population consisted of 64 patients with pathologically proved pancreatic ductal adenocarcinomas (PDACs), who underwent preoperative magnetic resonance imaging (MRI) examinations including diffusion-weighted imaging (DWI). The tumor ADCs were measured by two independent readers using six round ROIs with sizes ranging from 20 to 214 mm^2^ (9 to 97 pixels) in both the six separate measurements. The intra- and inter-observer variabilities were analyzed by using the coefficient of variance (CV), the interclass correlation coefficient (ICC) and Bland-Altman analysis. The mean ADCs measured with the 6 different-sized ROIs were compared using one-way repeated analysis of variance. The sample sizes were calculated by using 80% power and a 5% significance level to detect 10 to 25% changes in ADC measurements.

**Results:**

The largest ROI (ROI_214_) yielded the best intra-observer repeatability (CV, 6.3%; ICC, 0.93) and inter-observer reproducibility (CV, 10.1%; ICC, 0.84). The mean differences in ADC measurements ± limits of agreement between the two readers were (0.06 ± 0.47) × 10^−3^ mm^2^ for ROI_20_, (0.08 ± 0.46) × 10^−3^ mm^2^ for ROI_46_, (0.05 ± 0.37) × 10^−3^ mm^2^ for ROI_82_, (0.07 ± 0.42) × 10^−3^ mm^2^ for ROI_115_, (0.05 ± 0.43) × 10^−3^ mm^2^ for ROI_152_ and (−0.02 ± 0.29) × 10^−3^ mm^2^ for ROI_214_.

**Conclusions:**

ROI size had a considerable influence on the ADC measurements of PDACs.

## Background

Diffusion-weighted imaging (DWI) with quantitative measurement of apparent diffusion coefficient (ADC) values provide an alternative to conventional anatomical magnetic resonance imaging (MRI), such as T1- (T1WI) and T2-weighted imaging (T2WI), for the detection and characterization of pancreatic diseases. Descriptions of DWI techniques and their uses for pancreatic applications can be found in a review article by Barral et al. [1]. Notably, several recent studies focusing on quantitative measurements of ADC based on DWI have indicated that DWI is a valuable tool in the assessment of pancreatic cancer. Lots of studies reported that ADC values in pancreatic cancer were significantly lower compared with benign pancreatic tissue [2–17]. However, there is still a diagnostic challenge to the use of DWI in pancreatic cancer as described by Fukukura et al.,[13] and the reported ranges of ADC values for neoplastic tissues varies dramatically across different studies [2–17]. One reason for this observation is the large variation in the region of interest (ROI) sizes in these studies (ranging from 20 to 789 mm^2^), which may significantly influence ADC measurements [18, 19]. The avoidance of the placement of smaller ROIs within lesions is commonly recommended, particularly for response assessment studies [1, 20]. There is a clear need for the standardization of ROI sizes for ADC measurements of pancreatic cancers to enable the validation of this quantitative parameter as a qualified biomarker for longitudinal clinical trials. To our knowledge, the effect of ROI size on tumor ADC measurements in pancreatic cancer and the intra- and inter-observer variabilities have never been systematically studied. Thus, the aim of this study was to investigate the influences of ROI size on tumor ADC measurements and intra- and inter-observer variabilities in pancreatic cancer.

## Methods

### Patients

This single-center retrospective study was reviewed and approved by the ethics committee of our hospital, and informed consent was waived in this study. We undertook a review of our institution’s histopathological database of patients who underwent pancreatic resection between January 2014 and December 2014 and identified 280 consecutive patients who were diagnosed with pancreatic ductal adenocarcinoma (PDAC). Among these patients, 72 patients received preoperative MRI evaluations that included a DWI sequence within two weeks prior to surgery. We excluded 6 patients because the sizes of their tumors were insufficient to clearly indicate the effects of ROI size or draw the defined ROIs on the ADC map. Additionally, 2 other patients were excluded due to the presence of severe motion artifacts in their diffusion-weighted images. Thus, the final study population consisted of 64 patients (41 males; mean age: 61.1 ± 8.7 years; range: 40–78 years). The mean tumor size was 37 ± 9 mm (range, 21–70 mm).

### MRI procedure

All of the patients underwent upper abdominal MRI examinations (3.0-T, Signa HDxt, GE, Milwaukee, USA), including respiratory-triggered DWI weighted along three orthogonal gradient directions (b-values, 0 and 600 s/mm^2^). Fat saturation was performed with selective presaturation with inversion recovery. The main parameters and orders of MRI sequences are summarized in Table 1. Contrast-enhanced T1WI was performed with a gadopentetate dimeglumine injection (contrast media, 0.2–0.3 ml/kg; physiological saline, 10–15 ml).

**Table 1:** Parameters for MRI

Sequence	TR/TE (ms)	FOV (mm)	Matrix	Thickness/gap (mm)	Flip angle (°)	Slices	NEX	Bandwidth (kHz)	Speed factor
MRCP	7000/1253.4	300 × 300	288 × 288	64/0	-	6	0.92	31.2	-
T2WI	6316/73.8	360 ~ 400	320 × 192	5/1	90	20	2	83.3	2
DWI	6000/58.6	380 × 304	128 × 96	5/1	90	20	2/4^a^	250	2
T1WI	2.5/1.1	440 × 418	256 × 180	2.5/0	11	84	0.70	125	2

^a^NEX = 2 for DWI at b_0_, NEX = 4 for DWI at b_600_

*MRCP* magnetic resonance cholangiopancreatography, *T2WI* T2-weighted imaging, *DWI* diffusing weighted imaging, *T1WI* T1-weighted imaging

### Data analysis

**Fig. 1:**
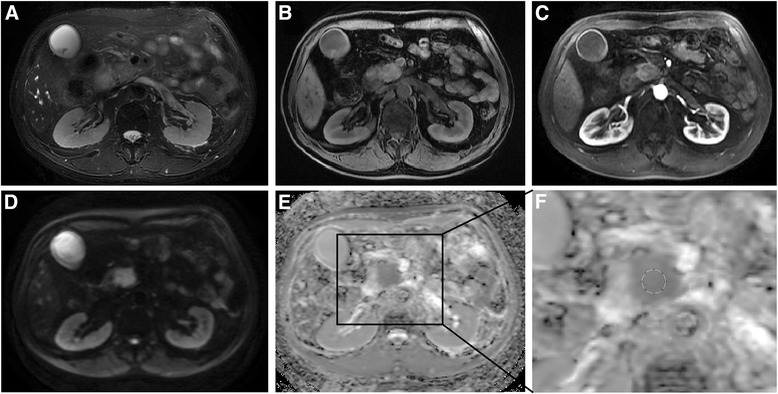
MRI images of histological proven pancreatic cancer in 64-year-old man. **a**: Axial T2WI; **b**: Axial precontrast T1WI; **c**: Axial contrast-enhanced arterial phase T1WI demonstrating the hypovascularity of the mass; **d**: DWI image (b = 600 s/mm^2^); **e**: ADC map; **f**: zoomed-in ADC map that indicates a ROI with a size of 214 mm^2^ (pixel number: 97) was used to measure the tumor ADC values

Based on the DWI images with b values of 0 and 600 s/mm^2^, ADC maps were calculated using a monoexponential model (ADC = (ln (SI_b0_/SI_b600_))/600) on a workstation (Function V9.4.05, AW 4.4, GE Healthcare). The ADC maps were reconstructed automatically with a 380 × 380 mm^2^ field of view (FOV) and a 256 × 256 matrix. The anonymous MR images of patients were sorted in a random order. The MRI data were evaluated and analyzed using ImageJ software (v1.47; http://rsb.info.nih.gov/ij). Six round ROIs (areas: 20, 46, 82, 115, 152, and 214 mm^2^; pixel numbers: 9, 21, 37, 52, 69, and 97) were drawn on the solid part of the tumor as identified on contrast enhanced T1WI (Fig. 1) while avoiding cystic lesion, dilated pancreatic ducts, and necrosis by referring to T2WI or T1WI images in both the six separate measurements. The mean ADC value and its standard deviation (SD) within each recorded ROI were obtained. All of the ADC values of the PDAC were independently measured by two observers (with 11 and 6 years of experience in abdominal radiology). To evaluate the intra-reader variability, the tumors ADCs were measured twice by reader 1 with an interval greater than three weeks. The first measurements of the tumor ADCs from reader 1 were further used to evaluate the inter-observer variability of the ADC values.

### Statistical analysis

SPSS (v16.0, SPSS Inc., Chicago, IL, USA) was used for the statistical analyses. Normality assumptions were assessed using the Shapiro-Wilk’s test. The intra- and inter-observer variabilities of tumor ADC measurements were evaluated with the interclass correlation coefficient (ICC) and Bland-Altman analysis [21, 22]. The measurement errors between the intra- and inter-observer measurements were quantified with the coefficient of variance (CV). Based on a two-sample unpaired t-test (80% power and a 5% significance level) [23, 24], the sample sizes needed to detect 10 to 25% changes in the ADC measurements for each ROI size were calculated. The homogeneity indices (defined by the SD/mean ADC within each recorded ROI) were also calculated for the tumors at each ROI size. The mean ADCs obtained from the 6 different-sized ROIs were compared by one-way repeated analysis of variance (ANOVA). *P* < 0.05 was established as the threshold for significance of the statistical analyses.

## Results

**Fig. 2:**
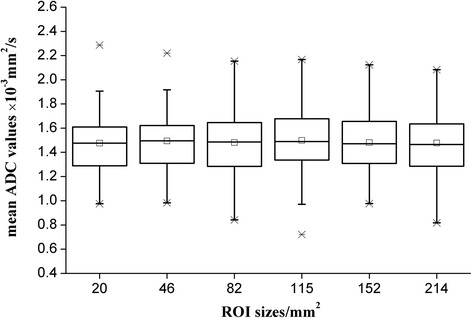
Box plots of the mean ADCs measured by the two readers with 6 round ROIs of sizes ranging from 20 to 214 mm^2^. The midline within each box represents the median value. The solid dot within each box represents the mean value. The crosses above and below each box indicate the minimum and maximum values, respectively

Typical MRI images and the ADC maps used for the ADC measurements are demonstrated in Fig. 1. As illustrated in Table 2, no significant difference was observed in the mean ADCs among the 6 different-sized ROIs for all the three measurements of two observers (all *P* > 0.05). Box plots providing graphic illustrations of the study data are presented in Fig. 2. The homogeneity index increased with increasing ROI size (Table 2).

**Table 2:** Mean ADC values (×10^−3^ mm^2^/s) and standard deviations (SDs) within each recorded ROI and the homogeneity indices (HI, calculated as the SD/mean ADC of each recorded ROI) of the pancreatic adenocarcinomas obtained with the 6 different-sized ROIs by two independent readers

	ROI_20_		ROI_46_		ROI_82_		ROI_115_		ROI_152_		ROI_214_		*P*-value^1^
	ADC	HI	ADC	HI	ADC	HI	ADC	HI	ADC	HI	ADC	HI	
Reader 1 (1^st^)	1.46 ± 0.28	0.07 ± 0.04	1.46 ± 0.27	0.11 ± 0.11	1.45 ± 0.27	0.12 ± 0.08	1.48 ± 0.26	0.13 ± 0.10	1.45 ± 0.26	0.14 ± 0.10	1.49 ± 0.25	0.15 ± 0.08	0.40
Reader 1 (2^nd^)	1.45 ± 0.28	0.07 ± 0.06	1.49 ± 0.25	0.09 ± 0.07	1.49 ± 0.26	0.10 ± 0.06	1.48 ± 0.26	0.12 ± 0.09	1.48 ± 0.25	0.13 ± 0.10	1.46 ± 0.26	0.14 ± 0.09	0.39
Reader 2	1.52 ± 0.27	0.06 ± 0.03	1.54 ± 0.25	0.08 ± 0.06	1.51 ± 0.26	0.10 ± 0.06	1.54 ± 0.29	0.12 ± 0.07	1.51 ± 0.27	0.13 ± 0.07	1.46 ± 0.28	0.15 ± 0.10	0.09

The data are summarized as mean ± SD; *ROI* region of interest, *ADC* Apparent diffusion coefficient, *HI* Homogeneity index

^1^One way repeated ANOVA of the mean ADC values from the 6 different-sized ROIs. Reader 1 (1^st^) and Reader 1 (2^nd^) represent the first and second measurements of the tumor ADCs from reader 1, respectively

### Intra-observer variability of ADC values

The bias, limit of agreement (LOA) and ICC values are summarized in Table 3. Good or excellent agreements (ICC > 0.78) were found for various ADC measurements with ROI_20_ (*i.e.* a ROI with an area of 20 mm^2^) to ROI_214_ between the two measurements from reader 1 (Table 3). The mean ADCs were more scattered with decreasing ROI sizes.

**Table 3:** Intra-observer reproducibility analysis of two repeated measurements of ADC by the same reader

	Mean ADC ± SD (×10^−3^ mm^2^/s)	CV	Bias	LOA	ICC
ROI_20_	1.45 ± 0.16	10.9%	−0.01	[−0.32, 0.30]	0.84
ROI_46_	1.47 ± 0.17	11.7%	0.03	[−0.31, 0.37]	0.78
ROI_82_	1.47 ± 0.13	9.1%	0.03	[−0.23, 0.30]	0.87
ROI_115_	1.48 ± 0.15	10.2%	<0.01	[−0.30, 0.29]	0.83
ROI_152_	1.47 ± 0.09	6.4%	0.02	[−0.16, 0.21]	0.93
ROI_214_	1.48 ± 0.09	6.3%	<0.01	[−0.19, 0.18]	0.93

*SD* standard deviation, *CV* coefficient of variance, *LOA* limit of agreement, *ICC* intra-class coefficient

### Inter-observer variability of ADC values

The bias, limit of agreement (LOA) and ICC values for the inter-observer comparison are summarized in Table 4. Similar to the intra-observer findings, the mean ADCs were more scattered with decreasing ROI sizes. The ICCs demonstrated moderate to excellent agreements from ROI_20_ to ROI_214_ between the ADC measurements from the two readers (ICCs, 0.59–0.84), and excellent agreement was only found for the ADC measurements with ROI_214_ between the two readers (ICC, 0.84; Table 4). The inter-observer agreements in the ADC measurements for each ROI were worse (i.e. smaller ICCs) than the intra-observer agreements.

**Table 4:** Inter-observer reproducibility of the two repeated measurements of ADC by two independent readers

	Mean ADC ± SD (×10^−3^ mm^2^/s)	CV	Bias	LOA	ICC
ROI_20_	1.49 ± 0.24	15.9%	0.06	[−0.40, 0.53]	0.63
ROI_46_	1.50 ± 0.24	15.7%	0.08	[−0.38, 0.54]	0.59
ROI_82_	1.48 ± 0.19	12.6%	0.05	[−0.31, 0.42]	0.76
ROI_115_	1.51 ± 0.21	14.0%	0.07	[−0.35, 0.48]	0.70
ROI_152_	1.48 ± 0.22	14.6%	0.05	[−0.37, 0.48]	0.66
ROI_214_	1.47 ± 0.15	10.1%	−0.02	[−0.31, 0.27]	0.84

*SD* standard deviation, *CV* coefficient of variance, *LOA* limit of agreement, *ICC* intra-class coefficient

### Sample size calculation

The sample size calculations for detecting 10 to 25% changes in the ADC values for the 6 sizes of ROIs are summarized in Table 5. The use of the largest ROI (214 mm^2^) for ADC measurements of PDACs as the end point required the smallest sample size, and the smaller ROIs required 56 to 150% more patients to detect similar changes.

**Table 5:** Sample size calculations for the 6 different-sized ROIs to detect 10 to 25% changes in ADC values

	CV (inter-observer)	10%	15%	20%	25%
ROI_20_	15.9%	80	36	20	13
ROI_46_	15.7%	78	35	19	12
ROI_82_	12.6%	50	22	13	8
ROI_115_	14.0%	62	28	15	10
ROI_152_	14.6%	67	30	17	11
ROI_214_	10.1%	32	14	8	5

*CV* coefficient of variance

## Discussion

ADC measurements have been utilized to investigate pancreatic adenocarcinomas; nevertheless, few studies have investigated the effect of ROI size on the reliability of ADC measurements in PDACs. In prior studies, ROI sizes for ADC measurements of PDACs have greatly varied [3, 4, 11–13, 17], and although no formal recommendations exist, a minimum size of 100 mm^2^ is commonly used [1]. ANOVA results show that the mean ADCs of tumors are independent of the ROI size in PDAC patients; however, ROI sizes have considerable influence on the intra- and inter-observer variabilities of ADC measurements of PDAC, regarding the inter-observer comparisons of the ADC values for the 6 ROIs, only the reproducibility of the mean ADC of the PDACs obtained from ROI_214_ was acceptable because the mean intra-observer biases of the ADC measurements did not exceed ± 0.10 × 10^−3^ mm^2^/s and the limit of agreement was less than ± 0.30 × 10^−3^ mm^2^/s (Tables 3 and 4) [23]. Additionally, excellent agreement between the two readers was found only in the ADC measurements from ROI_214_ (ICC, 0.84; Table 4). Compared with ROI_214_, ROI_115_ exhibited a 39% larger CV, and required 92% more patients to detect similar ADC changes in clinical trials.

We also found that the heterogeneities of the PDACs were evident and increased with larger ROIs. The homogeneity index was over 0.10 when the ROI size was larger than 82 mm^2^ (Table 2). Such heterogeneities led to measurement errors in the ADC values when small ROIs were used. It is noting, this study firstly assesses the influence of ROI size on the ADC measurements and intra- and inter-observer variabilities associated with PDAC and to provide sample size calculations for longitudinal studies.

Three ROI methods, including the whole-volume ROI [19, 25, 26], single-slice ROI [8, 10] and small solid-sample ROI [11–17] approaches have been used to obtain ADC measurements from tumors. In our previous studies, we found that despite its larger inter-observer variability [25], small solid-sample ROIs on tumors provide greater diagnostic performance in the assessment of PDAC compared with single-slice and whole-volume ROI methods [27],. In addition, it is difficult to perform ADC measurements with whole-volume or single-slice ROI method in many tumors of patients with PDAC because of the unclear boundaries of tumors on DWI images [13, 17, 25]. Solid-sample ROI is the most commonly used approach for ADC measurements of PDAC [2–7, 11–17]. The ROIs in the lesions are delineated to avoid pancreatic ducts, cystic lesions, and imaging artifacts. Therefore, the small solid-sample ROI approach was used in this study.

In addition to ROI methods, imaging acquisition parameters, including the field strength, respiratory compensation method, number of b values used, maximum b value selected, and post-processing approach, may influence tumor ADC measurements [28]. In our study, the DWI examinations were performed with a respiratory-triggered DWI sequence on a 3.0-T scanner in a single hospital, and thus bias produced by the use of different MRI machines or sequences was avoided. Kartalis et al. suggested that respiratory-triggered DWI is the recommended method for imaging a PDAC [29]. Additionally, the calculations of \ADC maps, based on DW images of b_0_ and b_600,_ which were selected to balance the image contrast and signal-to-noise ratio for pancreas, were performed by using a monoexponential model. Numerous DWI studies using similar b values for imaging pancreatic diseases [8, 10–12, 30–32]. Moreover, Koc Z and Erbay G reported that 600 s/mm^2^ is the optimal b-value for differentiation of abdominal lesions [15].

Our study had several limitations. First, longitudinal data are not available and the effect of ROI size on therapy response evaluation has not yet been investigated. Future larger scale longitudinal studies are required to confirm our results. Second, all of the examined lesions were pancreatic adenocarcinomas, and no other pathological pancreatic conditions were included in this study. Third, we enrolled patients who had undergone MRI examinations that were performed on the same 3.0-T system. This scenario may be ideal. The sample sizes acquired would likely increase in a multi-center taril using variable scanners due to greater CVs [33]. Finally, the effects of field strengths and selection of b-values on the ADC measurements were not evaluated; thus, field strengths and the selection of b-values may affect our results.

## Conclusions

This focused DWI study demonstrated that ROI size had a considerable influence on the ADC measurements of PDACs at 3.0-T. A large ROI size reduces the intra- and inter-observer variabilities in ADC measurements. A ROI size of 214 mm^2^ or greater will require a small sample size for future clinical trials.

## Abbreviations

ADC: Apparent diffusion coefficient
ANOVA: One-way repeated analysis of variance
Coefficient: Coefficient of variance
DWI: Diffusion-weighted imaging
FOV: Field of view
ICC: Interclass correlation CV
IQR: Interquartile range
LOA: Limit of agreement
MRI: Magnetic resonance imaging
PDAC: Pancreatic ductal adenocarcinomas
ROI: Region of interest
SD: Standard deviation
T1WI: T1-weighted imaging
T2WI: T2-weighted imaging
